# Investigation of Formulation and Process of Lyophilised Orally Disintegrating Tablet (ODT) Using Novel Amino Acid Combination

**DOI:** 10.3390/pharmaceutics2010001

**Published:** 2010-01-04

**Authors:** Farhan AlHusban, Amr M. ElShaer, Jiteen H. Kansara, Alan M. Smith, Liam M. Grover, Yvonne Perrie, Afzal R. Mohammed

**Affiliations:** 1 Aston Pharmacy School, Aston University, Birmingham B4 7ET, UK; 2 School of Chemical Engineering, University of Birmingham, Edgbaston, Birmingham B15 2TT, UK

**Keywords:** orally disintegrating tablets, proline, serine, lyophilisation, annealing, sublimation rate

## Abstract

Lyophilised orally disintegrating tablets (ODTs) have achieved a great success in overcoming dysphagia associated with conventional solid dosage forms. However, the extensive use of saccharides within the formulation limits their use in treatment of chronic illnesses. The current study demonstrates the feasibility of using combination of proline and serine to formulate zero sacharide ODTs and investigates the effect of freezing protocol on sublimation rate and tablets characteristics. The results showed that inclusion of proline and serine improved ODT properties when compared to individual counterparts. Additionally, annealing the ODTs facilitated the sublimation process and shortened the disintegration time.

## 1. Introduction

Development of solid oral dosage forms that disintegrate/dissolve rapidly in the mouth when in contact with saliva has attracted substantial attention in both academia and industry in order to address swallowing difficulties (dysphagia) associated with the conventional solid oral dosage forms (tablets and capsules) experienced by wide range of patient population [1]. These formulations are commonly referred to as orally disintegrating tablets (ODTs), orodispersible tablets, fast dissolving tablets, fast dispersing tablets, rapid dissolving tablets or fast melting tablets (FMTs). 

ODTs offer many advantages over other solid oral dosage forms. They can be easily swallowed (in contrast to conventional tablets and hard gelatin capsules) and can be used with patients who have difficulty in swallowing such as stroke victims, psychiatric, pediatric and geriatric patients [2]. ODTs also need no preparatory step prior to administration and therefore preferable when compared to extemporaneous suspension or effervescent granules. They have pleasant mouth feel and acceptable taste [3] and are preferred over the chewable tablets in which the bitter drug may leach during mastication [4]. Additionally, ODTs can be designed to provide fast onset of action by enhancing pre-gastric absorption through the buccal cavity, pharynx and oesophagus [2,5], and to increase the bioavailability by incorporating emulsions within the tablets [6,7].

Of the plethora of technologies available to fabricate ODTs such as three dimensional printing, moulding and direct compression, lyophilisation has been considered the most successful, as the resultant tablets have a highly porous structure, which permits rapid disintegration and hence easy swallowing. However one of the major drawbacks associated with freeze dried ODTs is the extensive use of saccharides and polyols in the formulation which limits there use in the treatment of chronic medical conditions and also for multiple dose medications primarily due to the limited allowable daily intake of these saccharides and polyols especially in pediatric, diabetic and obese patients. 

Research from our laboratory investigating the feasibility of using individual amino acids as matrix supporting/ disintegration enhancer agents in the formulation of lyophilised orally disintegrating tablets (data not published), has shown varied capability of amino acids to fulfill all the required characteristics for the formulation of lyophilised ODTs. For instance, proline showed complete wettability in water (disintegrating medium) with short wetting time, which is expected to improve the disintegration of ODTs; however, its inclusion in freeze dried formulations was limited due to the extremely low glass transition temperatures and consequently resulting in the collapse of the prepared formulations. On the other hand, serine based formulations displayed higher collapse temperature and produced elegant tablets even at high concentration, due to its tendency to crystallise in the frozen state, but was characterised by long disintegration time, which was explained by serine's partial wetting property, as the measured contact angel (θ) with water was 0º < θ < 90º. 

The main aim of the current research was to combine the benefits of proline and serine in the formulation of ODT with the aim to achieve a tablet with shorter disintegrating time (mainly due to the presence of highly wettable proline) and enhanced stability during freeze drying (due to the high glass transition and cryatllisation capacity of serine). The study investigated the influence of inclusion of various ratios of proline and serine at different total concentrations on the thermal properties of the frozen formulations, formation of intact tablets after freeze drying and ODT characteristics in terms of disintegration time and mechanical properties. The optimised formulation was then used to investigate the effect of freezing drying process parameters such as flash freezing and annealing on sublimation rate, disintegration time and mechanical properties of ODTs.

## 2. Results and Discussion

### 2.1. Thermal analysis and formation of intact tablets

The successful production of intact lyophilised tablets is totally dependant on the thermal profile of the frozen formulation and freeze drying conditions. The maximum tolerable product temperature during primary drying which ensures the formation of intact tablets, known as collapse temperature, can be estimated from the DSC profile of the frozen formulation. For amorphous formulations, the collapse temperature is usually 1 to 3 ºC higher than the glass transition [8].

At total amino acids concentration of 10% and 30% w/w (serine:proline combinations), all the studied combinations of serine and proline showed glass transition step (Tg’) in their heating scans at different temperatures depending on the total concentration and ratio of both amino acids (Table 1). The inclusion of these two amino acids in the formulation had a plasticising effect in the formulation as increasing the total concentration of the amino acids significantly lowered the Tg’ temperature. However, proline had a higher plasticising effect on the system than serine since a gradual increase in proline ratio within the formulations was associated with a steady decrease in Tg’ values. For example, at a total concentration of 30% (w/w), increasing proline ratio from 0 to 45 to 100 decreased the Tg’ from -25.66 ± 0.01 to -32.26 ± 0.1 to -37.65 ± 0.24 ºC, respectively (Table 1). Estimation of the collapse temperatures for 10% w/w amorphous formulations suggested the presence of a high safety margin between the shelf and collapse temperature which resulted in the formation of intact and smooth tablets. On the other hand, formulations at 30% w/w total concentration of the combined amino acids did not reveal any morphological deterioration despite the small difference between the glass transition and shelf temperature. The possibility of any micro collapse for these formulations cannot be ruled out.

**Table 1:** Glass transition temperatures (^o^C) of maximally freeze concentrate solutions of 5% gelatin after inclusion combinations of proline and serine at total concentration of 10% and 30% w/w. Values are represented as mean ± standard deviation (*n* = 3).

Combination (proline:serine)	Total concentration (w/w)	
	10%	30%
0:100	-18.63 ± 0.05	-25.66 ± 0.01
15:85	-19.12 ± 0.11	-27.97 ± 0.12
30:70	-19.71 ± 0.09	-29.52 ± 0.42
45:55	-20.35 ± 0.21	-32.26 ± 0.10
70:30	-20.87 ± 0.16	-34.24 ± 0.10
85:15	-21.31 ± 0.08	-35.57 ± 0.07
100:0	-21.47 ± 0.12	-37.65 ± 0.24

**Figure 1:**
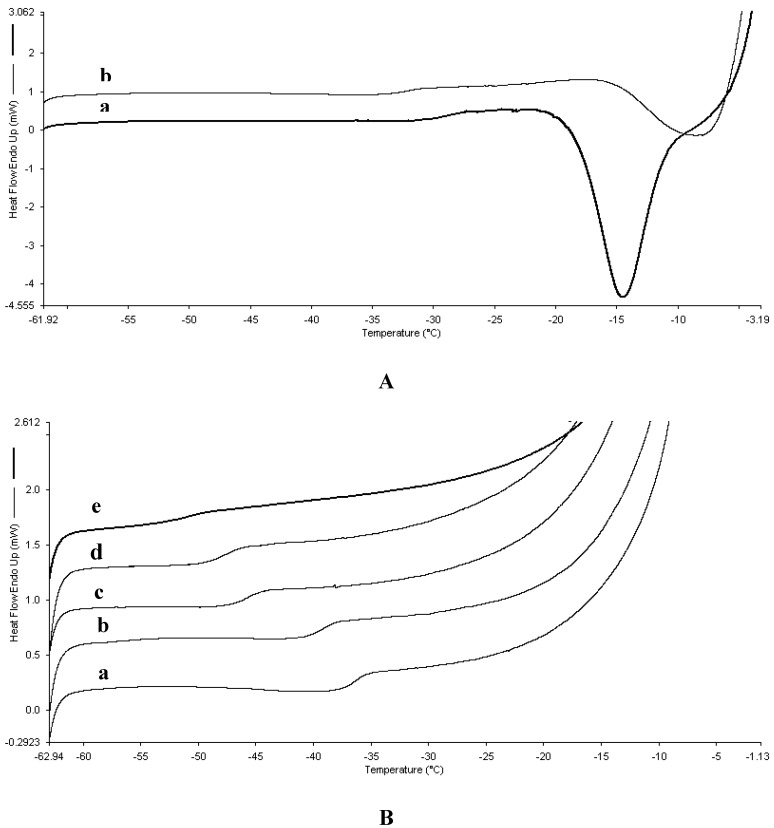
**A.** Overlaid DSC heating curves of frozen formulations that exhibited serine crystallisation at total concentration 50% amino acid. (a) serine to proline ratio of 100:0. (b) Serine to proline ratio of 85:15. **B.** Overlaid DSC heating curves of frozen formulations that did not show tendency to crystallize at total concentration of 50% amino acids. (a) 70:30 (serine: proline); (b) 55:45 (serine: proline); (c) 70:30 (serine: proline); (d) 85:15 (serine: proline); (e) 0:100 (serine: proline).

DSC analysis of formulations with total concentration of 50% and 70% w/w (total solid) of combinations of proline and serine are presented in Figure 1 and Table 2, respectively. At these high concentrations, formulations containing serine only displayed crystallisation event during their heating scans. Inclusion of small amount of proline, 15:85 (proline:serine), seemed to drift the crystallisation temperature of serine to a higher temperature when compared to serine alone formulation (Figure 1A). Further increase in proline ratio within the formulations inhibited serine crystallisation completely and the trend continued as observed in 10% and 30% w/w formulations which was evident by lowering of the glass transition temperature (Figure 1B and Table 2). Freeze drying of these formulations was less efficient when compared to 10% and 30% w/w formulations which can be explained by higher concentration of proline that decreases the glass transition and inhibits serine crystallisation. As a result, all the formulations with Tg’ less than -40 ºC collapsed and therefore no tablet was formed. Freeze drying of such formulations is possible by decreasing the shelf temperature of the cycle, but it is associated with significant increase in the primary drying time. It has been shown previously that lowering the shelf temperature by 5 °C may result in increase in the primary drying time of about 15 hours [9].

**Table 2:** Glass transition temperatures of maximally freeze concentrate solutions (Tg’) and crystallisation temperatures of frozen solutions of 5% gelatin after inclusion of combinations of proline and serine at total concentration of 70%. Values are represented as mean ± standard deviation (*n* = 3).

Proline/Serine ratio	Tg’ (^o^C)	Crystallization temperature (^o^C)
0:100	*	-23.99 ± 0.53
15:85	-33.13 ± 0.43	-16.62 ± 0.95
30:70	-39.54 ± 0.32	-14.02 ±1.08
45:55	-44.91 ± 0.64	*
70:30	-51.44 ± 2.27	*
85:15	-57.63 ± 0.97	*
100:0	>65	*

### 2.2. Characterisation of ODTs

#### 2.2.1. Mechanical properties

**Figure 2:**
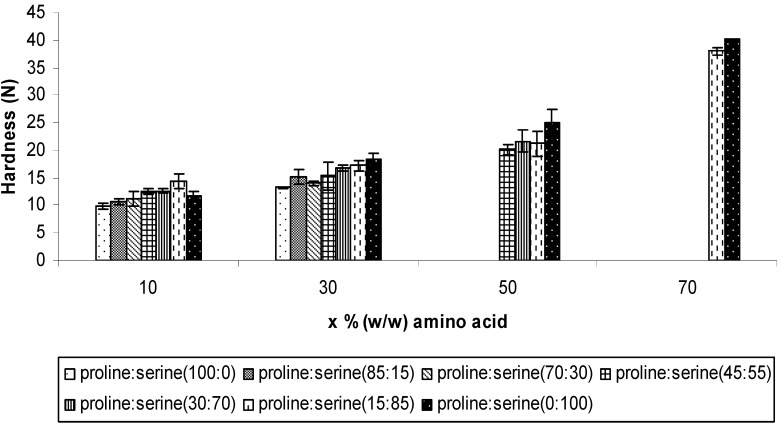
The hardness (Newton) of the ODTs after inclusion combinations of proline and serine at total concentrations of 10%, 30%, 50%, and 70% w/w. Values are represented as mean ± standard deviation (*n* = 3).

**Figure 3:**
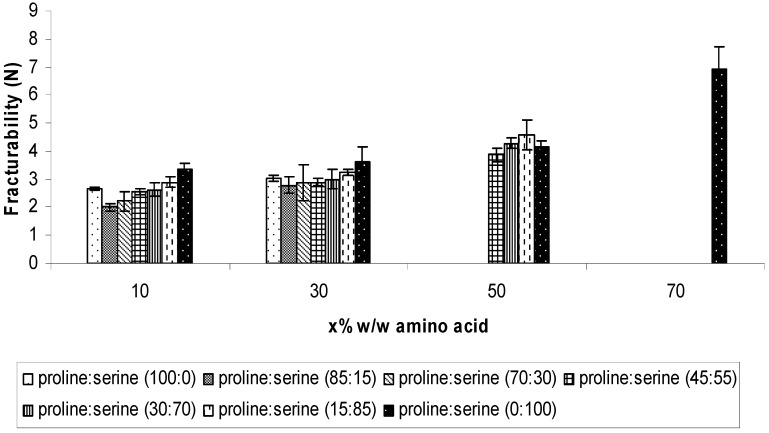
The fracturability (Newton) of the ODTs after inclusion combinations of proline and serine at total concentrations of 10%, 30%, 50%, and 70% w/w. Values are represented as mean ± standard deviation (*n* = 3).

All the successfully freeze dried formulations were characterised in terms of mechanical properties by measuring their resistance to compression by a 5 mm diameter probe (hardness) and penetration by a 1 mm diameter probe (fracturability). The influence of the total amino acids concentration and proline to serine ratio within the formulation on the hardness and fracturability of ODTs are presented in Figure 2 and Figure 3, respectively. The results showed that the hardness of the ODTs was significantly improved by inclusion of a higher total concentration of both amino acids (one way ANOVA/Tukey-Kramer: ρ < 0.05). For instance, each increment in the total concentration of 15:85 of proline:serine formulation was associated with a significant increase in the hardness, from 14.46 ± 1.33 N at concentration of 10% to 17.24 ± 0.92 N at 30% w/w, to 21.29 ± 2.26 N at 50% and then to 37.96 ± 0.68 N at concentration of 70% (one way ANOVA/Tukey-Kramer: ρ < 0.05). However, at the same total concentration, combinations with higher serine ratio provided stronger tablets compared to tablets with high proline ratio suggesting better capability of serine to enhance the hardness of lyophilised ODTs (Figure 2). For example, at total concentration of 10%, increasing serine ratio from zero to 55% resulted in significant improvement in the ODTs hardness from 9.85 ± 0.41 N to 12.47 ± 0.5 N (one way ANOVA/Tukey-Kramer: ρ < 0.05) and then to 14.47 ± 1.3 N (one way ANOVA/Tukey-Kramer: ρ < 0.01) by further increase serine ratio to 85% w/w of the total amino acids.

The ODTs fracturability results are presented in Figure 3. Statistical analysis of the data showed that increasing the total amino acids concentration from 10% to 30% did not improve the fracturability. However, significant improvements were achieved by increasing the total concentration to 50% (one way ANOVA/Tukey-Kramer: ρ < 0.01) or 70% (one way ANOVA/Tukey-Kramer: ρ < 0.001). Also, the results showed no particular influence of changing the ratio of proline and serine within the formulation. Accordingly, the results suggested that the fracturability was mainly influenced by the total concentration of the amino acids rather than the ratio of proline to serine within the formulation.

#### 2.2.2. Disintegration time of the ODTs

**Figure 4:**
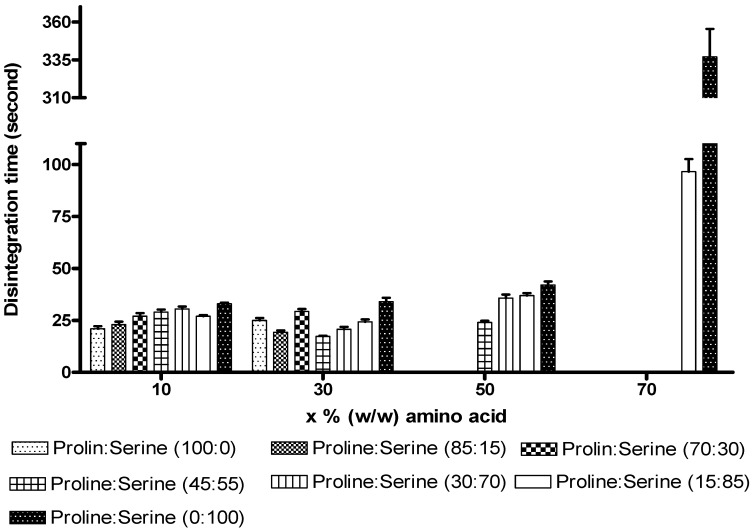
The disintegration time (seconds) of the ODTs after inclusion combinations of proline and serine at total concentrations of 10%, 30%, 50%, and 70% w/w. Values are represented as mean ± standard deviation (*n* = 3).

The disintegration time profile of the tablets is presented in Figure 4. At a total concentration of 10%, tablets containing proline only achieved the shortest disintegration time of 21.0 ± 2.1 s (*n* =3). Upon gradual increment in serine ratio, the disintegration times increased steadily to 29.0 ± 2.2 s for the 45:55 of proline:serine formulation and then to 33.0 ± 1.0 s for tablets with serine only. At a total amino acids concentration of 30% w/w, the shortest disintegration time was 17.3 ± 0.6 s for the 45:55 of proline:serine combination. It was anticipated that formulations with higher proline ratio (higher than 45%) would achieve the shortest disintegration time but because of their narrow freeze drying safety margin, invisible partial micro collapse ( as discussed in the section on thermal properties) might have deteriorated their disintegration profile. Formulations with high freeze drying safety margin, which contained proline ratio less than 45%, confirmed this theory by following the expected trend where longer disintegration time was associated with any increase in serine ratio (Figure 4).

The successfully freeze dried tablets based on total concentrations of amino acids of 50 and 70% followed the expected trend and the shortest disintegrations at both concentrations were recorded by formulations with the highest ratio of proline (Figure 4).

These results can be explained depending on the mechanism of disintegration of ODTs. Generally, the fast disintegration profile of lyophilised ODTs is attributed to the highly porous structure that allows fast diffusion of water (disintegrating medium) through highly wettable matrixes, which disintegrate/dissolve rapidly upon contact with water [10]. In the current formulations, inclusion of higher concentration of proline is expected to increase the wettability of the matrix while increasing total concentration of the amino acids reduces the total porosity of the tablets. Accordingly, a balance between the wettability and porosity is required to achieve short disintegration time. The current results (Figure 4) suggest that 45:55 combination of proline:serine at a total concentration of 30% achieved best balance between wettability (containing 13.50% proline) and porosity with total amino acid concentration of 30% w/w (intermediate concentration) which consequently achieved the shortest disintegration time in the study (17.3 ± 0.6 s). It is interesting to note that that even small intervention in this balance can lead to significant deterioration in the disintegration time. For example, formulations with higher porosity (lower total concentration of amino acids) but slightly lower wettability (lower concentration of proline), as in tablets based on proline only at total concentration of 10% (of total tablet weight), displayed significantly longer disintegration time. Similarly, formulations with higher wettability but smaller porosity, as in tablets based on 45:55 of proline:serine at total concentration of 50%, did not achieve shorter disintegration time (Figure 4). Similar trend was observed from a previous study reported from our laboratory investigating the influence of saccharides and polyols on disintegration time where a parabolic relationship was noticed within optimal concentration (30–40% w/w) of matrix supporting/disintegration enhancing agents [11].

#### 2.2.3. Lyophilised tablet index

The tablet characterization results (hardness and disintegration time) have shown that serine to proline ratio and their total concentration in the formulation have contrasting influence on the mechanical properties and disintegration time of the lyophilised ODTs. Therefore, the overall tablets properties were evaluated depending on a single parameter that integrates the hardness and disintegration time of ODTs, called lyophilised tablet index (LTI) [12]. LTI is calculated by dividing the measured hardness by the disintegration time of certain ODT formulation which means the higher value the better the overall properties (high hardness and low disintegration time). The results (Table 3) proved that combining proline and serine as matrix supporting /disintegration enhancing agents in the formulation creates ODTs with superior overall properties (disintegration time and harness) than using proline or serine individually. The highest LTI value was 0.88 for the formulation with 45:55 of proline:serine at total concentration of 30% (LTI= 0.88), followed by the same combination of proline and serine but at concentration of 50% with LTI value of 0.82, suggesting that the deterioration in the disintegration, caused by increasing the total concentration from 30% to 50%, was more profound than the improvement in the hardness. 

**Table 3:** The lyophilised tablet index values of The ODTs varied concentration of proline and serine combinations.

Combination (prolin:serine)	Total concentration (w/w)			
	10%	30%	50%	30%
100:0	0.47	0.51	-	-
85:15	0.46	0.78	-	-
70:30	0.41	0.48	-	-
45:55	0.43	0.88	0.84	-
30:70	0.41	0.82	0.61	-
15:85	0.54	0.71	0.58	0.39
0:100	0.44	0.54	0.59	0.12

### 2.3. The influence of freezing protocol on the primary drying rate and ODTs characteristics.

In the freeze-drying process, the freezing step is one of the most important steps as it determines the size and morphology of the ice crystals within the frozen material and, consequently, the final inner-structural feature of the freeze-dried material [13]. Thus, in lyophilised tablets, the freezing protocol is expected to influence not only the freeze drying process (sublimation rate and primary drying time) [14] but also tablet characteristics after freeze-drying (the disintegration time and mechanical properties). In this study, three freezing protocols; freezing at -80 °C using pre-cooled shelves with or without annealing at -20 °C for 12 hours and flash freezing using liquid nitrogen, were investigated for their effects on the sublimation rate, inner-structural features of the freeze dried tablets and tablets characteristics of the formulation with the highest LTI value (45:55 of proline: serine at total concentration of 30% w/w).

Mercury porosimetry was used to investigate the structure of the freeze dried tablets, because it preserves the internal morphology of the sample during the measurement, does not require cutting the tablets which may alter the cake structure and also gives consistent estimation of the pore size and pore size distribution for the whole tablets. 

#### 2.3.1. Influence on primary drying rate

**Figure 5:**
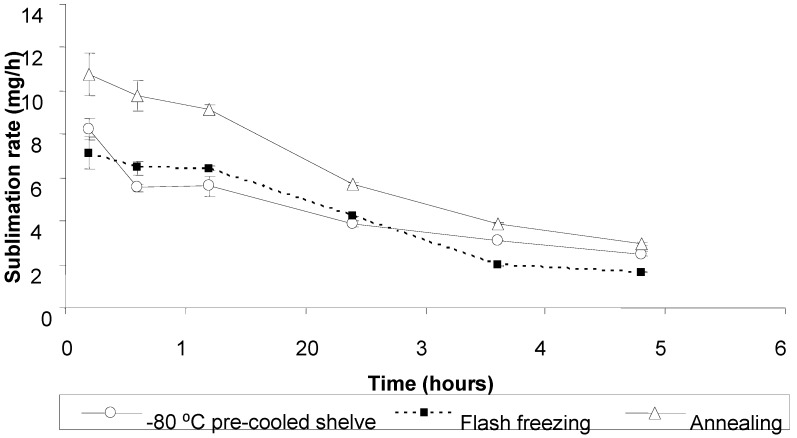
Sublimation rate (mg/h) as a function of time for the ODT formulation with 45:55 of proline: serine at total concentration of 30% w/w frozen by various methods. Values are represented as mean ± standard deviation.

Figure 5 shows primary drying rates of tablets based on 45:55 of proline: serine at total concentration of 30% w/w after applying different freezing methods. At all time points the average drying rates of the annealed tablets were significantly higher than tablet frozen using -80 °C pre-cooled shelves or liquid nitrogen (flash freezing), both without annealing (one way ANOVA/Tukey-Kramer: ρ < 0.01). Moreover, the decrease in drying rate with time due to increasing the thickness of the dried layer seemed to be steady and consistent for the annealed tablets compared to the tablets without annealing. Given that all the tablets were of a similar formulation and freeze dried under the same conditions, all the differences in the primary drying profiles are attributed to the inner morphology of the tablets imposed by the freezing regime.

**Figure 6:**
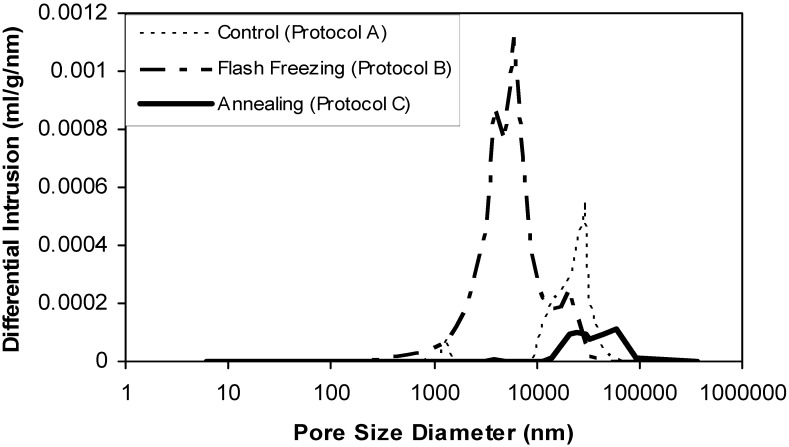
Pore size distributions of ODTs prepared using different drying protocols, including: freeze drying, flash freezing and annealing.

The mercury porosimetry data (pore size distribution) are presented in Figure 6. The results showed that flash freezing produced tablets with the smallest modal pore diameter (6 µm), but with a broad pore size distribution between 284 nm to 30 µm. When the formulation froze at -80 ˚C, the pores exhibited a larger modal diameter (30 µm) with pores in the range 1 to 60 µm. On the other hand, annealed tablets exhibited the largest pores with a modal diameter of 60 µm distributed from 13 to 370 µm. 

In case of flash freezing and pre-cooled shelves at -80 ˚C, freezing at lower temperature and faster rate resulted in a larger number of dispersed minute ice crystals, and consequently smaller pores after freeze drying. These small pores create narrow and complex channels for water vapor removal during the sublimation and therefore higher mass transfer resistance (MTR), which in turn decreases the sublimation rate [15]. Moreover, the lack of direct control over ice nucleation temperature using these freezing methods resulted in wide pore size distributions (Figure 6) and hence heterogeneous MTR values, which is translated as inconsistent decrease in the average primary drying rate over time (Figure 5) [16]. 

Annealing, on the other hand, is a known technique to enhance the growth of ice crystals and eliminate the initial variation in crystal size distribution by a phenomena known as Ostwald ripening, where smaller ice crystals melt quickly and then merge with larger crystals (not completely melted) as a result of raising the temperature above Tg’, whereas re cooling the sample fixes the structure of the large crystals [17,18,19]. Thus, the annealed tablets in this study exhibited larger mean pore diameter with narrower size distribution compared to the tablets without annealing (Figure 6). These structural features facilitated and homogenised water-vapor transmission (low and constant MTR) and therefore high and consistent primary drying rates were achieved. 

The current results are consistent with previous studies [13,14,15,20], where adding annealing to the freezing regime has enhanced ice crystals growth and, consequently, increasing the sublimation. Other researchers have employed different physical approaches to enhance and control ice crystals growth, with the aim of reducing primary drying times, including ultrasounds [21], vacuum induced surface freezing [22] and high electrical field [23].

#### 2.3.2. Influence on ODT characteristics

We have demonstrated above that the structure of the freeze dried cake had changed significantly when applying different freezing protocols. These morphological changes can directly influence the basic properties of the lyophilised formulation. For ODTs, the disintegration time and mechanical properties are the key aspects to investigate.

**Figure 7:**
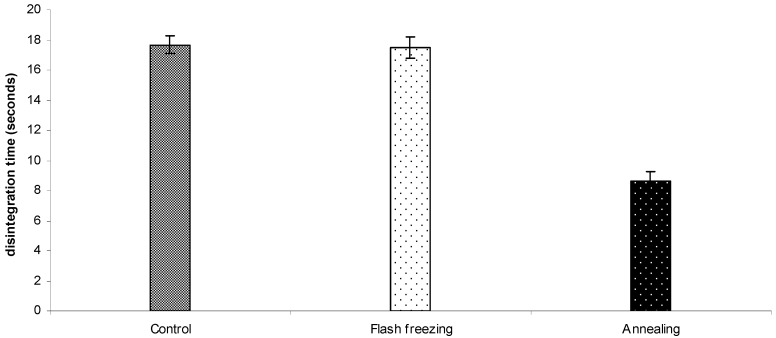
Disintegration time of ODTs based on 45:55 of proline: serine at total concentration of 30% w/w after applying different freezing protocols. Values are represented as mean ± standard deviation (*n = 3*).

The effect of different freezing protocols on the ODTs disintegration time is presented in Figure 7. The results revealed that the annealed tablets had significantly shorter average disintegration time of 8.6 ± 0.6 s when compared to 17.5 ± 0.5 s and 17.3 ± 0.6 s for tablets frozen using liquid nitrogen and at -80 °C Pre-cooled shelve, respectively, (one way ANOVA/Tukey-Kramer: ρ < 0.001). The fast disintegration of the annealed tablets can be attributed to their large pores (Figure 6) that facilitate rapid diffusion of water (the disintegrating medium).

**Figure 8:**
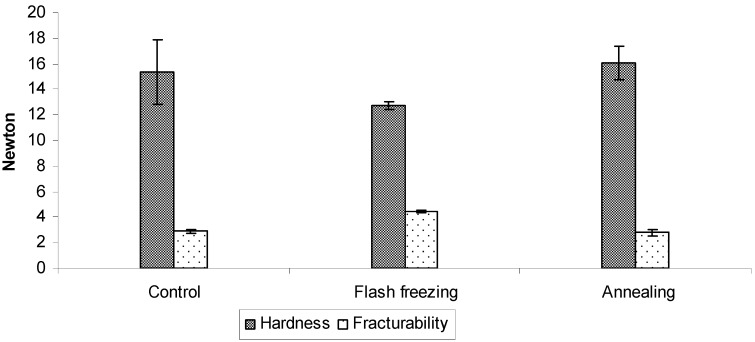
Hardness and fracturability of ODTs based on 45:55 of proline:serine at total concentration of 30% w/w after applying different freezing protocols. Values are represented as mean ± standard deviation (*n = 3*).

**Figure 9:**
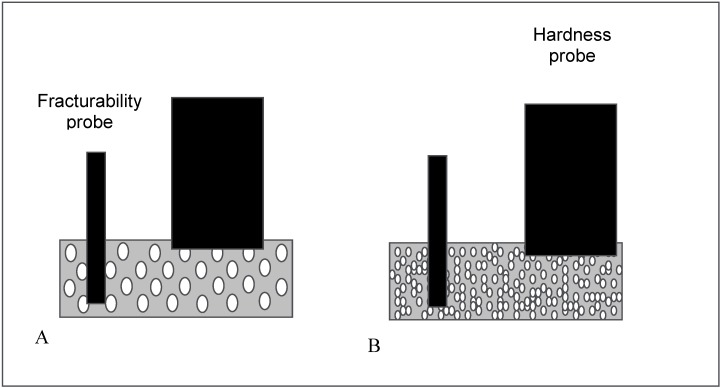
Schematic representation of the effect of freezing protocol on hardness and fracturability of ODTs. **A.** tablets frozen for 2 hours at -80 °C precooled freezer, annealed at -20 °C for 12 hours and then transferred back to -80 °C freezer. **B.** tablets flash frozen using liquid nitrogen.

The hardness and fracturability of the ODTs are presented in Figure 8. The effect of annealing on the mechanical properties of the ODTs was not significant when compared to ODTs frozen at -80 °C pre-cooled shelve without annealing, statistically, there was no difference in terms of their hardness nor fracturability (ρ > 0.05). However, the results showed that flash freezing of the formulation using liquid nitrogen significantly modified the mechanical properties of the tablets, as lower hardness of 12.7 ± 0.3 N was recorded compared to 16.0 ± 1.3 N for the annealed ODTs (ρ < 0.05) but with significantly higher fracturability (4.4 ± 0.1 N compared to 2.8 ± 0.1 N for the annealed, P < 0.05). Thus, the organised larger pores structure of the annealed tablets seems to have stronger resistant for the compression by the hardness probe (5 mm diameter) but weaker resistant toward penetration of the thin probe (1 mm diameter) that measures the fracturability, compared to tortuous and smaller pores structure of the flash frozen ODTs (Figure 9). 

## 3. Experimental Section

### 3.1. Materials

Gelatin from bovine skin, type B (Bloom strength ~ 75), _L_-Proline (C_5_H_9_NO_2_, Reagent plus^TM^ ≥ 99%), _L_-Serine (C_3_H_7_NO_3_, Reagent plus^TM^ ≥ 99%), were all purchased from SIGMA^®^, USA. All the materials were used as received.

### 3.2. Methods

#### 3.2.1. Formulation of ODTs to investigate the effect of _L_-proline and _L_-serine combination on the tablets characteristics

Various ratios (100:0, 85:15, 70:30, 45:55, 30:70, 15:85, 0:100) of _L_-proline and _L_-serine at total concentrations of 10%, 30%, 50%, and 70% w/w (total solid) were added to 5% (w/w) gelatine stock solution. 1.5 g of the prepared solution was transferred to a PEG mould, frozen at -80 °C for 2 hours and then freeze-dried (ADVANTAGE Freeze-dryer, VIRTIS) according to an optimized regime (primary drying for 48 hours at a shelf temperature of -40 °C and secondary drying for 10 hours at a shelf temperature of 20 °C and vacuum of 50 mTorr. All the formulations (28 different formulations) were prepared in triplicate from three independent batches. From each batch 3 tablets were freeze dried and characterised for disintegration time, hardness and fracturability. In total 252 tablets were prepared (28 X 3 X 3).

#### 3.2.2. The influence of freezing protocol on the primary drying rate and ODTs characteristics

The formulation with the best performance in terms of disintegration time and mechanical properties from the previous study (2.2.1) was used to investigate effects of freezing protocols on the sublimation rate and tablets characteristics. The following three freezing protocols were applied:

Protocol 1:
  The formulation was frozen in -80 °C freezer.
Protocol 2 (flash freezing):
  The formulation was immersed in liquid nitrogen for 40 seconds then kept at -80 °C freezer.
Protocol 3 (annealing):
  The formulation was frozen at -80 °C precooled freezer for 2 hours, annealed at -20 °C precooled freezer for 12 hours and then transferred back to -80 °C freezer.

The sublimation rate was studied by freeze drying samples (from each protocol) at shelf temperature of -40 °C, condenser temperature of -80 °C and 55 mTorr vacuum. Samples were withdrawn from the freeze dryer at predetermined time intervals (2, 6, 12, 24, 36 and 48 hours) and the amount of water sublimed was evaluated using weight difference method. All the measurements were done in triplicate of independently prepared samples.

In order to study the effect of freezing protocol on tablet characteristics, nine samples from each protocol entered a complete freeze drying cycle using similar regime used in section (2.2.1).

#### 3.2.3. Differential scanning calorimetry

Differential scanning calorimeter (Pyris Diamond DSC) was used to investigate the glass transition temperatures (Tg) and the crystallization events of the frozen formulations. 10–15 mg of the liquid formulation was transferred into an aluminium pan (50 µL capacity) and then sealed with an aluminium top. The sample was cooled to -65 ºC and then heated to 20 ºC at 5 ºC/min. To determine the glass transition temperature of the maximally freeze concentrate sample (Tg’), after initial cooling to -65 ºC, annealing for 10 min at temperature of 2 ºC higher than the relevant glass transition temperature (Tg) was added before carrying out the above method. Nitrogen was used as a purge gas at a flow rate of 20 mL/min. Indium and zinc were used to calibrate the heat flow and melting point onset (melting point: 156.6 °C, ∆Hm: 28.42 J/g for Indium and melting point: 419.47 °C ∆Hm: 108.26 J/g for Zinc). The obtained thermograms were analysed using Pyris Manager Software (version 5.00.02) where Tg and Tg' values were determined from the intersection of relative tangents to the baseline. The experiment was performed in triplicate and an empty aluminium pan was used as a reference cell for all the measurements.

#### 3.2.4. Texture analysis

In order to investigate the fracturability and hardness of the prepared tablets, QTS 25 texture analyser (CNS Farnell, Hertfordshire, UK) was used. Fracturability was studied by using 1 mm diameter penetration probe which penetrates 4 mm of the tablet at a speed of 6 mm/min and the peak force was measured in Newton (N) after 3 mm of penetration. The tablet hardness was measured using a 5 mm diameter compression probe which compresses the tablets to 2 mm depth at a speed of 6 mm/min and the peak force is measured in Newtons after 1 mm compression. The obtained data was analysed by TexturePro software. All fracturability and hardness measurements were performed in triplicate for each formulation and the data is presented as mean ± standard deviation.

#### 3.2.5. *In vitro* disintegration study of the tablets

Disintegration time is the time required for ODTs to disintegrate completely without leaving any solid residue. *In vitro* disintegration time for lyophilised ODTs was evaluated using US pharmacopoeia monograph (<701> disintegration). 

Erweka ZT3, Appartebau, GMBH was used in this study as a disintegration apparatus and distilled water (800 mL) as disintegration medium; the disintegration medium temperature was maintained at 37 °C by thermostat. At each time, one tablet was placed in the basket rack assembly and covered by transparent plastic disk. The disintegration time was taken as the time required for ODTs to disintegrate completely without leaving any solid residue. All the measurements are carried out six times and presented as (mean ± standard deviation). 

#### 3.2.6. Mercury porosimetry

Mercury porosimetry was used to evaluate the influence of processing on the pore size distribution of the resulting tablets. Measurements were made using an Autopore IV 9500 mercury porosimeter (Micromeritics, UK). Samples were stored overnight in a vacuum to remove moisture and were then weighed and loaded into a 5 cc bulb 1.190 ml stem, penetrometer (Micromeritics, UK). Measurements of pore size distribution were made in the low and high pressure chambers of the porosimeter to provide the pore size distribution in the range 6 nm to 360 μm. The resulting measurements of intrusion volume (ml/g/nm) were used to calculate pore size distribution.

#### 3.2.7. Statistical analysis

Graph Pad Instat® software was used for the statistical analysis study. Data groups were compared using one way analysis of variance (ANOVA) and pair-wise multiple comparisons method (Tukey-Kramer multiple comparison test). Standard deviation (SD) was used to report the error in the figures and texts. Probability values of 95% (P < 0.05) were used to determine the significant difference.

## 4. Conclusions

This study has demonstrated that inclusion of optimised combinations of serine and proline in the formulation of lyophilised orally disintegrating tablets can combine the benefits of high wettability and stability resulting in the formation of tablets with superior properties over that of individual amino acids. The inclusion of serine in the formulation at high concentration enhances the mechanical properties of the ODTs without compromising the formation of intact tablets. On the other hand, proline promotes the disintegration by enhancing the wettability of the ODTs. Annealing induces morphological changes in the ODTs that not only allow faster sublimation rate but also shorter disintegration time.
